# Adenomyosis and fibrosis define the morphological memory of the postpartum uterus of dairy cows previously exposed to metritis

**DOI:** 10.3168/jdsc.2024-0633

**Published:** 2024-10-30

**Authors:** Isabella Sellmer Ramos, Monica O. Caldeira, Scott E. Poock, Joao G.N. Moraes, Matthew C. Lucy, Amanda L. Patterson

**Affiliations:** 1 Division of Animal Sciences, University of Missouri, Columbia, MO 65211; 2 College of Veterinary Medicine, University of Missouri, Columbia, MO 65211; 3 Department of Animal and Food Sciences, Oklahoma State University, Stillwater, OK 74075; 4 Department of Obstetrics, Gynecology and Women's Health, University of Missouri, Columbia, MO 65212

## Abstract

Optimal fertility after calving in lactating dairy cows is dependent upon the successful completion of uterine involution. Invasion of pathogenic bacteria into the uterine environment within the first week postpartum can lead to uterine disease (metritis). Metritis is associated with decreased fertility and a failure or delay in establishing pregnancy. We hypothesized that early postpartum metritis would be associated with long-term changes in uterine morphology that begin within the first 30 d postpartum (DPP) and are present during a typical breeding window (2–6 mo postpartum). First parity Holstein cows were diagnosed with metritis (M) or deemed healthy (H) at 7 to 10 DPP and uterine tissues were collected and analyzed postmortem at 30 (experiment [Exp.] 1; M = 10, H = 10), or 80 and 165 (Exp. 2; M = 9, H = 10) DPP for the presence of abnormal morphology, including abnormal invasion of endometrial glands and stroma into the myometrium (adenomyosis) and endometrial fibrosis. Glands were identified using immunohistochemistry for FOXA2 (uterine gland specific marker) and fibrosis was identified using Masson's trichrome stain (MTS). The severity of adenomyosis was assessed by the number and size of adenomyotic foci and the distance of foci from the endometrium-myometrium interface (EMI). The degree of fibrosis was defined by MTS intensity. Data were analyzed using a 2-way ANOVA procedure including the effect of metritis and DPP on the dependent variables foci size, distance from EMI, and MTS intensity. A negative binomial regression model was used for the dependent variable of foci number. The presence, size, and distance from the EMI of adenomyotic foci were greater for later postpartum (Exp. 2, 80 and 165 DPP) and early postpartum cows (Exp. 1, 30 DPP) that were previously diagnosed with metritis, suggesting greater severity of adenomyosis. Endometrial fibrosis was greater at the stratum basalis (near EMI) compared with the stratum compactum endometrium (near uterine lumen) for all Exp. 2 (80 and 165 DPP) cows. Greater fibrosis (regardless of endometrial region) was observed in cows diagnosed with metritis compared with healthy controls. Taken together, these data indicate that early postpartum metritis is associated with long-term changes to postpartum uterine morphology, including increased pathological fibrogenesis, leading to the presence of late postpartum endometrial fibrosis (scar tissue), and aberrant endometrial invasion into the myometrium (adenomyosis). Additionally, increased collagen fiber at the EMI suggests a correlation between the development of adenomyosis and fibrosis, which could result from sustained endometrial inflammation caused by uterine disease.

## Highlights

• Metritis was associated with abnormal uterine morphology (fibrosis and adenomyosis).
• Metritis increased the number, diameter, and invasion of adenomyotic lesions.
• Metritis was associated with greater endometrial fibrosis at mid lactation.
• Endometrial fibrosis was greatest at the myometrial border.

## Graphical Abstract

**Figure:**
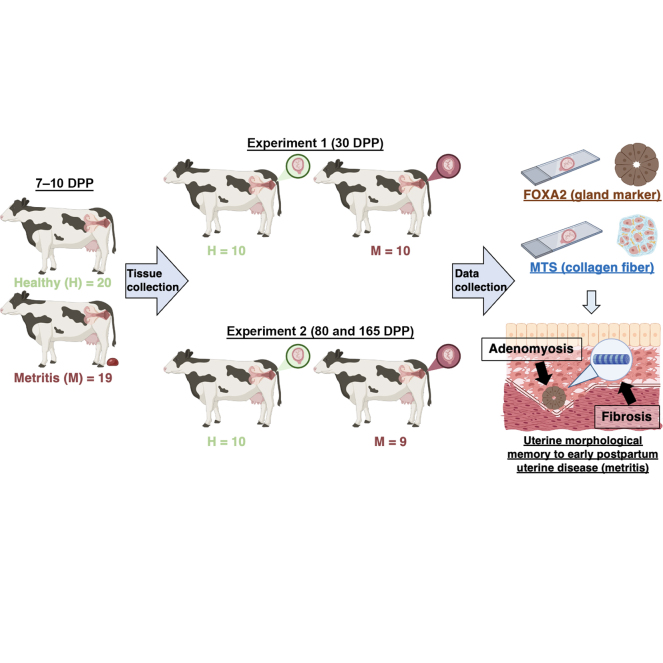
**Summary:** We investigated the possibility that early postpartum metritis was associated with the development of aberrant uterine morphology at early (30 days postpartum) and mid lactation (80 and 165 days postpartum) to explain reduced fertility at breeding in cows with metritis. Cows with metritis had increased severity of adenomyosis (endometrial gland invasion into the myometrium) and increased endometrial fibrosis (scar tissue) at mid lactation. Endometrial fibrosis was greater in the endometrial-myometrial interface (endometrium closest to the myometrium) of cows that had metritis, indicative of abnormal tissue repair in response to previous damage. We concluded that metritis was associated with the development of adenomyosis and fibrosis, likely due to a pathological reparative response to early postpartum uterine disease, suggesting the presence of a morphological memory of the uterus during uterine involution that is sustained through the breeding period. Created with BioRender.com.

A cascade of physiological, morphological, and metabolic adaptations during the transition from pregnancy to parturition and subsequent lactation creates challenges for dairy cows after calving. Successful completion of early postpartum events is important for early postpartum milk production and optimal reproductive performance during the breeding period later postpartum (Lucy, 2019; Sheldon et al., 2019). Among these events is the process of uterine involution, characterized by the functional re-establishment of the uterine histoarchitecture to support subsequent early embryonic development and the attachment of the conceptus to the endometrial epithelia (Buch et al., 1955; Sheldon, 2004).

Despite the success achieved by reproductive programs, fertility in lactating dairy cows remains suboptimal due to factors contributing to early embryonic mortality (Ribeiro et al., 2016; Ribeiro and Carvalho, 2017). One of these is factors is metritis, an early postpartum uterine disease affecting approximately 25% of lactating dairy cows (LeBlanc, 2023; Bruinjé et al., 2024). Metritis is caused by dysbiosis of pathogenic bacteria within the uterus postpartum (Galvão et al., 2019; Pinedo et al., 2020). Failure to resolve the bacterial infection and associated inflammation can delay or permanently affect the re-establishment of the uterine histoarchitecture, potentially explaining lower fertility in cows that recover from metritis (Sellmer Ramos et al., 2023).

Inflammation fosters the development of benign uterine disorders, which pose implications for female fertility. Among these disorders is a condition known as “adenomyosis,” characterized by the abnormal invasion of glandular epithelia surrounded by endometrial stroma into the muscular layer of the uterus (myometrium). Adenomyosis is an important gynecological disorder in women due to its significant impact on fertility (AlAshqar et al., 2021). Previous studies have reported the presence of adenomyosis in dairy cattle and its positive relationship with both postpartum uterine disease and endocrine dysfunction (Korzekwa et al., 2013; Talebkhan Garoussi et al., 2015). The mechanisms that regulate the development of adenomyosis are not completely understood, but its prevalence among uteri that have sustained injuries suggests a potential bridge between chronic uterine inflammation and fertility losses in dairy cows. We hypothesized that early postpartum metritis would be associated with a sustained uterine morphological memory that included aberrant injury responses such as the abnormal migration of endometrial stroma and glandular epithelia into the myometrium (adenomyosis) and uterine fibrosis in dairy cows. Such findings could shed light on additional factors that challenge the successful establishment of pregnancy during the breeding period.

Study procedures were approved by the University of Missouri Institutional Animal Care and Use Committee (protocol number 9635). This is a retrospective study using samples from our previously published reports in which we investigated uterine gland morphology, gene expression, and microbiome of the postpartum dairy cow uterus (Sellmer Ramos et al., 2023; Silva et al., 2024). The sample size for the current study was based on logistical constraints (e.g., retrospective analysis of previously collected uterine samples) as well as previous experience with similar studies in postpartum cows from our laboratory (referenced above) (Moore et al., 2017). Details of the cows used for this study are as follows. For experiment (**Exp.**) 1 and 2, first parity Holstein cows were selected from a large confinement herd in eastern Kansas or the University of Missouri herd. Cows with a clinical diagnosis of metritis (fetid, red-brown, watery vaginal discharge with a flaccid uterus) at 7 to 10 DPP were selected and matched with clinically healthy postpartum cows (viscous [not watery] and nonfetid discharge) that calved during the same week (Sheldon et al., 2006). For Exp. 1, Kansas herd cows were moved to the University of Missouri shortly after diagnosis, and cows from both herds were slaughtered at approximately 30 DPP (29.1 ± 1.7 DPP; mean ± SD [n = 10 metritic; n = 10 healthy]).

Cows in Exp. 1 were also used for a previous study in which metritis and healthy cows were randomly assigned to either antibiotic treatment (healthy; n = 5 and metritis, n = 5; ceftiofur hydrochloride [i.m.; 2.2 mg/kg for 3 d]) or not treated (healthy, n = 5 and metritic, n = 5). Results from that experiment demonstrated no long-term effect of antibiotic treatment on various study endpoints, including uterine gene expression, microbiome, and inflammation (Caldeira et al., 2023). Cows in Exp. 2 were treated with ceftiofur hydrochloride at the discretion of the herdsman, as described previously (Silva et al., 2024). For Exp. 2, cows from the Kansas herd remained in the herd until transport to the University of Missouri 1 d before slaughter at approximately 80 DPP (n = 5 metritis and n = 5 control; 79.0 ± 7.5 DPP; mean ± SD) or approximately 165 DPP (n = 4 metritis and n = 5 control; 165.0 ± 4.9 DPP; mean ± SD).

Cows were slaughtered by captive bolt and exsanguination at the University of Missouri abattoir. The reproductive tract was removed, wrapped in a sterile surgical drape, placed on ice in a plastic bag, and transported to the laboratory. The uterine lumen was flushed with sterile saline and a cross-section from the middle portion of the nongravid horn that included both caruncular and intercaruncular areas was fixed in 10% neutral buffered formalin. Tissues were then paraffin embedded and subjected to immunohistochemistry and Masson's trichrome staining (**MTS**). Immunohistochemistry for FOXA2 (a marker of uterine glands) was performed on 5 µm sections according to the previously reported protocol (Sellmer Ramos et al., 2023; 1:500 dilution; catalog number ab108422, Abcam, Waltham, MA) to identify adenomyotic foci in uterine cross-sections. Foci of adenomyosis were defined by the presence of FOXA2 positive glandular epithelia surrounded by endometrial stroma within the myometrial layer. An entire uterine cross-section per cow was surveyed for the presence of adenomyotic foci using a Leica DM 4000 B microscope (Leica, Wetzlar, Germany) at 100× magnification. To assess the severity and progression of adenomyosis, the following morphological measurements were made for each foci identified per uterine section for each cow: (1) number of foci, (2) width and height mean diameter of foci (log_10_), and (3) foci-to-endometrium-myometrium interface (**EMI**) distance. Foci diameter and distance (µm) were collected using the scale measurement tool within the LAS X software (LASX v1.7, Leica).

The MTS was performed to assess endometrial fibrosis in sections within a 20 µm distance of the FOXA2-assayed sections using a kit (catalog number ab150686, Abcam) according to the manufacturer's instructions. Due to the scarcity of evident positive stain for collagen fiber in Exp. 1 (3 out of 20 cows), the quantification of endometrial fibrosis was limited to Exp. 2. The endometrium cross-section was divided into 2 anatomical regions, defined as the stratum basalis (endometrial stroma region closest to the myometrium) and stratum compactum (endometrial stroma region closest to the uterine lumen). Two photographs were captured of each anatomical region for one cross-section per each individual cow at 200× magnification using the Leica DM 4000 B microscope fitted with a Leica DFC 450C camera. The intensity of collagen fiber was quantified by calculating the mean pixel intensity of collagen fiber staining (blue stain) using the linear measurement tool in ImageJ (Java 1.8.0_172, NIH, Bethesda MD). To control for variance across tissue sections that can occur during the staining procedure, a background staining intensity measurement was collected from the myometrial region (smooth muscle fibers stained red), and the mean intensity of collagen fiber staining was obtained by subtracting the collagen quantification from background stain.

Data were analyzed using SAS 9.4 (SAS Institute Inc., Cary, NC). For Exp. 1 and Exp. 2, the dependent variables were (1) the number of myometrial adenomyotic foci within a uterine cross-section, (2) the mean width and height diameter (µm) of adenomyotic foci, and (3) the foci-to-EMI distance (µm). For Exp. 2, an additional dependent variable was added: (4) the mean pixel intensity of the stain for endometrial collagen fiber (indicative of fibrosis). Dependent variable 1 was analyzed using a negative binomial regression model that included status and days postpartum, due to the lack of a normal distribution (zero-inflated and right-skewed). The dependent variables 2 and 3 were analyzed using a 2-way ANOVA procedure (PROC GLM) of SAS. A model that included status (metritis or healthy) and days postpartum (30, 80, or 165 DPP) and all interactions were normally distributed and fit. Data from Exp. 1 (30 DPP) and Exp. 2 (80 or 165 DPP) were analyzed collectively to determine if early postpartum cows (30 DPP) or later postpartum cows (80 or 165 DPP) differed in number, diameter, and distance of adenomyotic foci in each respective uterine cross-section. Dependent variable 4 was analyzed using a 2-way ANOVA procedure (PROC GLM) of SAS, in a model that included data from Exp. 2 (80 and 165 DPP) and the fixed effects of disease status, DPP, and endometrial layer. Regardless of the analysis, the LSM were separated by using the Tukey procedure. Significance was declared at *P* < 0.05. A statistical tendency was 0.05 < *P* < 0.10. Data are presented as LSM ± SEM unless stated otherwise.

The FOXA2 expression (Figure 1A) and MTS (Figure 1B) were used to identify adenomyotic foci. There were fewer (chi-squared = 7.6; *P* < 0.01) cows with adenomyotic foci in Exp. 1 (30 DPP; 7 of 20 cows [35%]) compared with Exp. 2 (80 and 165 DPP; 15 out of 19 cows [79%]). When stratifying by disease status (metritis or healthy, Figure 1C–J) at 30 DPP (Exp. 1), the presence of adenomyosis was evenly distributed between healthy (4 out of 10, Figure 1C, E) and metritis cows (3 out of 10, Figures 1G, I). However, for Exp. 2, all metritis cows had adenomyosis (80 DPP, 5 out of 5; 165 DPP, 5 out of 5; Figure 1H, J), whereas approximately half of the healthy cows had adenomyosis (80 DPP, 3 out of 5; 165 DPP, 2 out of 4; Figure 1D, F).

**Figure 1:**
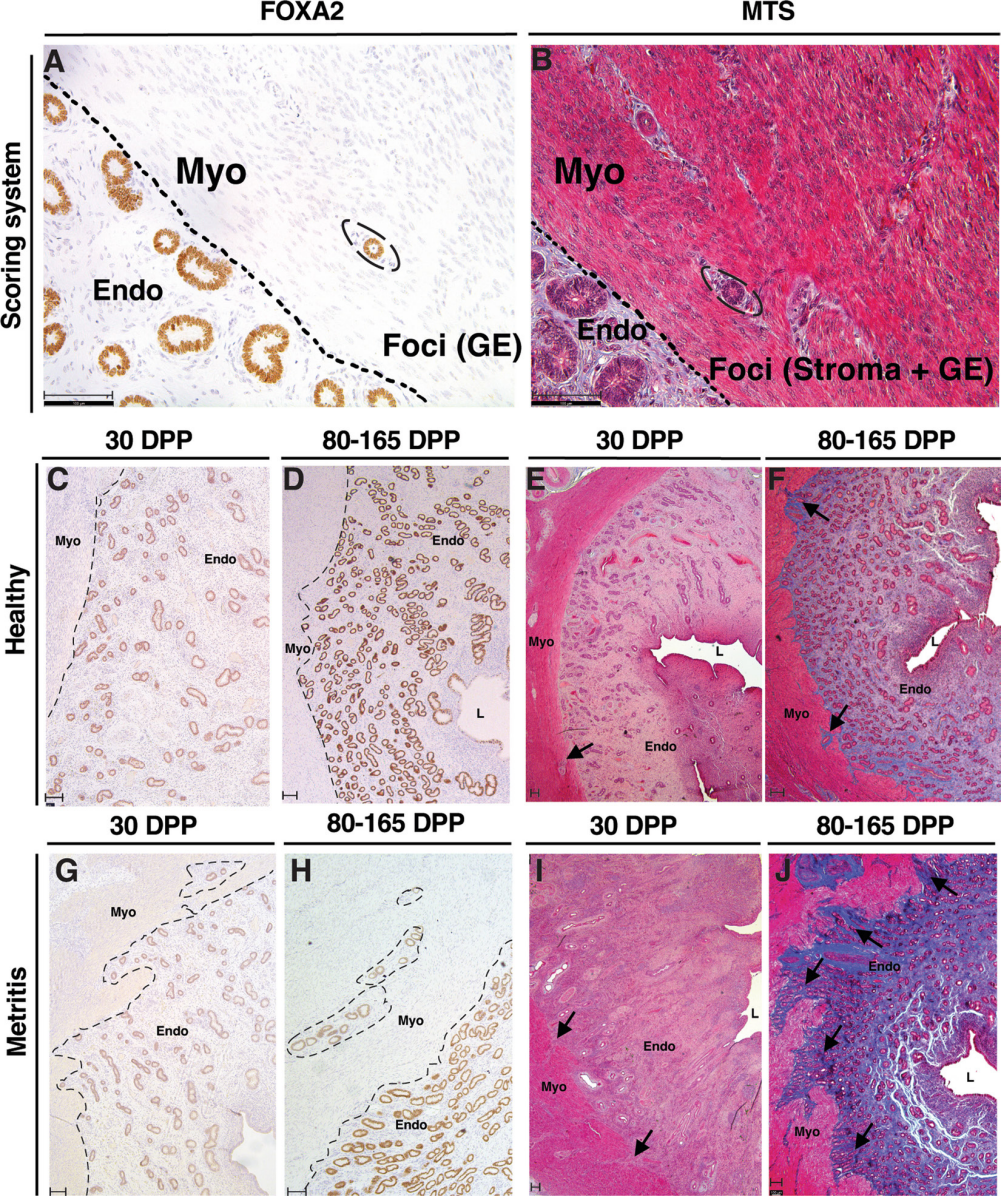
Uterine histological cross-sections of healthy (C, D, E, F) and metritic (G, H, I, J) cows at early (Exp. 1, 30 DPP; C, E, G, I) and late (Exp. 2, 80 and 165 DPP; D, F, H, J) days postpartum (DPP) using FOXA2 (gland marker; A, C, D, G, H) and Masson's trichrome stain (MTS; connective tissue stain; B, E, F, I, J) as a method to identify the presence of adenomyotic lesions in the myometrium (40× magnification; A, B) and to assess the severity of myometrial invasion by endometrial stroma and glandular epithelium (2.5× magnification; C–J). Myo = myometrium; Endo = endometrium; L = uterine lumen; GE = glandular epithelium; Foci = adenomyotic foci. Bar = 100 μm.

There was an effect of DPP on the number of adenomyotic foci per uterine cross-section and an effect of DPP on the diameter of adenomyotic foci (Table 1). The total number of adenomyotic foci per uterine cross-section was greater in cows at 80 or 165 DPP compared with 30 DPP (Table 1; *P* < 0.001). There was no effect of DPP on foci diameter or the EMI distance.

**Table 1:** Summary of LSM for adenomyosis foci diameter, foci-to-endometrium-myometrium interface (EMI) distance, and foci number for days postpartum and disease status

Variable	LSM	SE	95% CI	
			Lower bound	Upper bound
Foci number				
Status†				
Healthy	0.57	0.30	−0.02	1.16
Metritis	1.25	0.26	0.74	1.76
Days postpartum, d				
30	−0.16^a^	0.32	−0.79	0.47
80	1.41^b^	0.34	0.74	2.08
165	1.48^b^	0.35	0.79	2.17
Foci diameter				
Status				
Healthy	2.32^a^	0.05	2.22	2.42
Metritis	2.49^b^	0.04	2.41	2.57
Days postpartum, d				
30	2.35	0.06	2.23	2.42
80	2.51	0.05	2.41	2.61
165	2.34	0.06	2.22	2.46
Foci-to-endometrium distance				
Status				
Healthy	205.7^a^	63.4	81.44	329.96
Metritis	383.6^b^	50.3	285.01	482.19
Days postpartum, d				
30	264.9	71.9	123.98	405.82
80	334.1	64.3	208.07	460.13
165	285.1	73.7	140.65	429.55

a,b Means with different superscripts are significantly different between groups by status (healthy, metritis) or by days postpartum at *P* < 0.05.

† Metritis greater than healthy at *P* < 0.10.

There was an effect of disease status (metritis or healthy) on the adenomyotic foci-to-EMI distance (*P* = 0.034) and an effect of disease status on foci diameter (*P* = 0.028; Table 1). The distance and diameter were greater in cows diagnosed with metritis when compared with healthy cows. Last, there was a tendency (*P* < 0.08) for a greater number of adenomyotic foci per uterine section in metritis compared with healthy cows (Table 1). Collectively, these results suggest an association between early postpartum metritis with the severity of adenomyosis (foci-to-EMI distance, foci diameter, and number of foci).

There was little to no detectable collagen fiber with the MTS at 30 DPP (Exp. 1) regardless of disease status (metritis or healthy; Figure 1E, I). For Exp. 2 (80 and 165 DPP), there was an effect of both disease status and endometrial layer independently on the intensity of endometrial fibrosis (*P* < 0.001). The mean pixel intensity of blue collagen fiber stain was greater in the stratum basalis (near EMI; 107.6 ± 3.3; Figure 2B, E) when compared with the stratum compactum (near lumen; 72.5 ± 3.3; *P* < 0.001; Figure 2C, F), and was greater in the endometrium of cows that were diagnosed with metritis (103.1 ± 3.2; Figure 2D) compared with healthy cows (77.0 ± 3.4; *P* < 0.001; Figure 2A) at both 80 and 165 DPP. There was also an interaction between disease status and endometrial layer (*P* < 0.05). The greatest intensity of collagen fiber stain was observed in the stratum basalis of cows that had metritis (128.0 ± 4.5) compared with the same layer of cows that were healthy (87.2 ± 4.8; *P* = 0.004).

**Figure 2:**
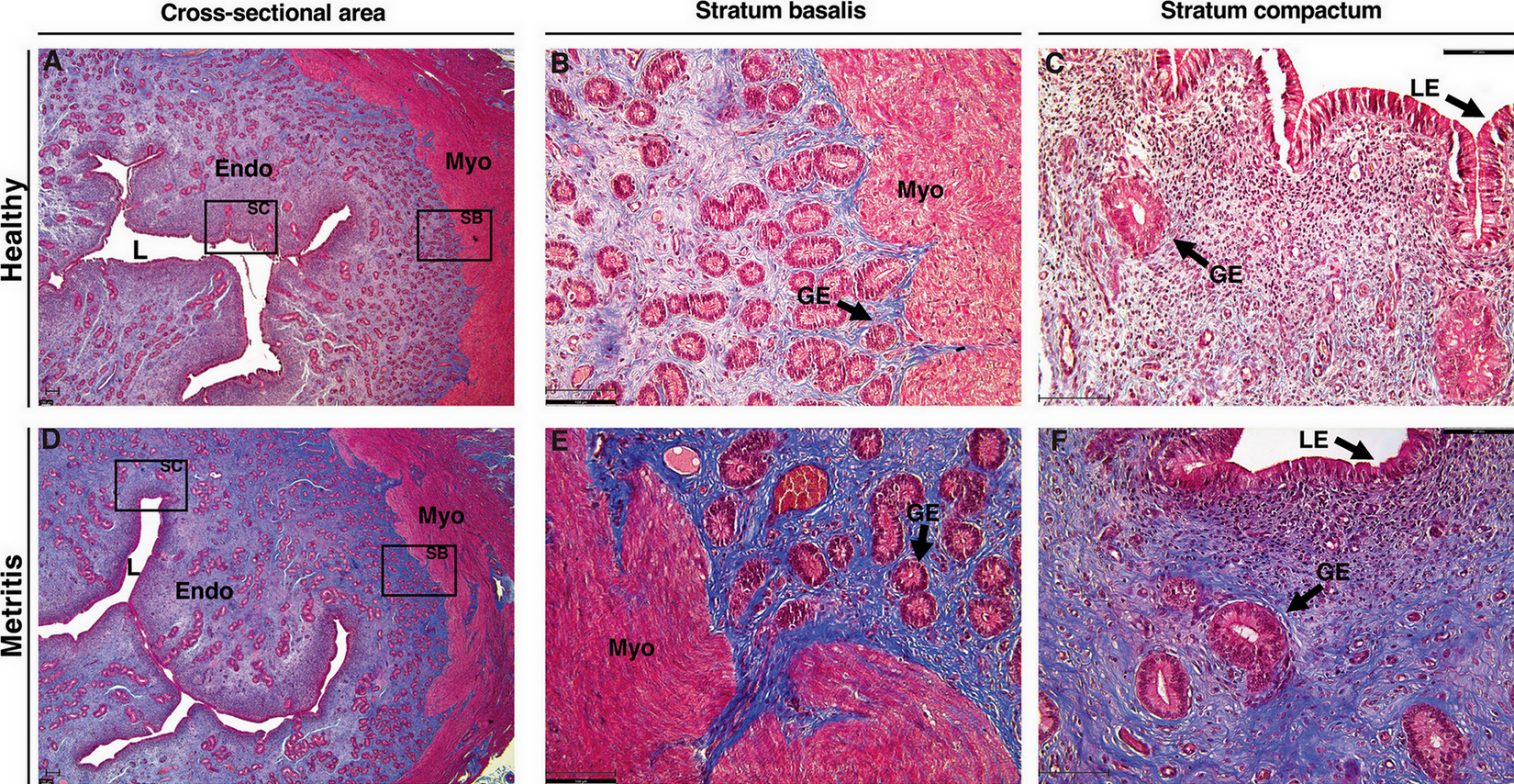
Endometrial cross-sections of late postpartum (Exp. 2; 80 and 165 DPP) cows stained with Masson's trichrome to detect and quantify collagen fiber (blue stain) within the stratum basalis (SB; endometrial stroma closest to the myometrium [Myo]; B, E) and stratum compactum (SC; endometrial stroma closest to the luminal epithelium [LE]; C, F) layers of the endometrium (Endo) of healthy (A, B, C) and metritic (D, E, F) cows as a method to assess the severity of postpartum uterine fibrosis. Insets in A and D correspond to images B and C, and E and F, respectively. Myo = myometrium (red stain); L = uterine lumen; GE = glandular epithelium; scale bar = 100 μm.

This series of observations demonstrates the negative effect that metritis has on the re-establishment of normal uterine histoarchitecture. We observed that the uterus of cows diagnosed with metritis between 7 to 10 DPP had a greater invasion of endometrial glands into the myometrium (adenomyosis). The pathogenesis of adenomyosis is hypothesized to be initiated by the disruption of the endometrial-myometrial border, followed by the subsequent invasion of endometrial tissue (glandular epithelia surrounded by stromal fibroblasts) into the myometrium (Bulun et al., 2021; Li et al., 2024). Local trauma at the endometrial-myometrial interface (e.g., parturition followed by uterine infection) associated with myometrial hyperperistalsis leads to the activation of the tissue injury-repair mechanism (**TIAR**), which is marked by pro-inflammatory signaling (Leyendecker et al., 2009; Scull et al., 2010). Chronic endometrial inflammation, particularly when induced by secreted cytokine activity, has been demonstrated to promote endometrial infiltration and migration (Bodur et al., 2015). Accordingly, we demonstrated in a series of in vitro studies using 80 and 165 DPP-derived bovine endometrial epithelial cells that LPS upregulated the expression of pro-inflammatory markers, such as *TNF, CXCL8, PTGS2*, and *SAA3* and genes involved in extracellular matrix (**EXCM**) organization and cell-cell adhesion, including *COL1A1, COL1A2, MMP2, MMP9*, and *TGFBI* (Caldeira et al., 2023). These novel findings provide the first evidence of an epithelial immunological memory within the uterus of lactating dairy cows following a diagnosis of metritis and association with the abnormal invasion of endometrial glands and stroma into the myometrium.

We found greater fibrosis (collagen fiber staining) in the endometrium of later postpartum cows (Exp. 2, 80 and 165 DPP) compared with the cows at 30 DPP (Exp. 1; Figure 1). In the context of TIAR, fibrogenesis is a biological process that results from TIAR-induced inflammation, which consequently induces EXCM synthesis and deposition upon fibroblast activation (Wynn, 2007). Recent studies investigating the uteri of women who previously sustained endometrial injury reported a link between adenomyotic lesions and fibrosis. Chronic myometrial hyperperistalsis led by insult to the endometrial-myometrial interface has been associated with muscle fiber disruption in focal adenomyotic areas, which subsequently triggers a repeated tissue injury and repair response (Guo and Groothuis, 2018; Kobayashi et al., 2021) resulting in epithelial-to-mesenchymal transition, fibroblast-to-myofibroblast trans-differentiation, and subsequent fibrogenesis (Liu et al., 2016; Xiao et al., 2020; Yildiz et al., 2023). Moreover, the presence of fibroblast subtypes with aberrant gene expression was identified in a recent single-cell RNA-seq study that aimed to characterize the cellular landscape of adenomyotic lesions of women (Niu et al., 2023). Accordingly, we observed that the intensity of fibrosis in later postpartum cows (Exp. 2, 80 and 165 DPP) was increased in cows that were diagnosed with metritis, and more evidently in the stratum basalis (deep) endometrium nearest the myometrium. In addition to fibroblast differentiation, TIAR-induced fibrogenesis can be exacerbated by the presence of chronic inflammation, more notably attributed to the presence of macrophages and regulatory T cells (Bacci et al., 2009). Based on joint metataxonomics and global endometrial transcriptome, we recently demonstrated that the early postpartum metritis microbiome of Exp. 2 (80 and 165 DPP) cows modulated the endometrial transcriptome at mid lactation and suggested the presence of residual immune cells within the uterus of cows that were diagnosed with early postpartum uterine disease (Silva et al., 2024). Such findings indicate a timeline for the development of fibrotic-associated endometrial invasion and could explain why little to no fibrosis was observed in Exp. 1 (30 DPP) cows (Figure 1).

Given the nature of this study (e.g., retrospective sample analysis), our sample size and number of uterine samples per animal were limited. Additionally, enrollment of Exp. 1 and Exp. 2 subjects occurred in consecutive years. Last, the role that retained placentae and dystocia play in the development of adenomyosis was unable to be assessed in the current study. Given that, we recognize such factors introduce limitations to our study and require further exploration.

Taken together, our data bring novel insights into the multimodal effect of metritis on the fertility of lactating dairy cows. We demonstrated that metritis was associated with abnormal endometrial migration at the time of uterine involution (Exp. 1, 30 DPP), and aberrant uterine morphology was sustained for a long period, indicating an association with long-term changes in the normal uterine histoarchitecture (Exp. 2, 80 and 165 DPP). Collectively, our results suggest that metritis modulates the re-establishment of a functional uterine landscape, perhaps due to chronic inflammation leading to abnormal endometrial migration and the presence of nonfunctional tissue (fibrosis). In corroboration, a recent study demonstrated that women with adenomyosis had a greater incidence of chronic endometritis compared with women without adenomyosis (Khan et al., 2021), indicating an association between endometritis and adenomyosis in women. In sum, we hypothesize that the presence of a morphological memory to factors that negatively affect tissue repair could play an important role in the fertility of dairy cows at the uterine level and requires further investigation.
